# Comparison of NEMA characterizations for Discovery MI and Discovery MI-DR TOF PET/CT systems at different sites and with other commercial PET/CT systems

**DOI:** 10.1186/s40658-020-0271-x

**Published:** 2020-01-14

**Authors:** Alexandre Chicheportiche, Rami Marciano, Marina Orevi

**Affiliations:** Department of Nuclear Medicine and Biophysics, Hadassah Hebrew University Medical Center, 91120 Jerusalem, Israel

**Keywords:** NEMA, PET/CT, Physical performance, Discovery MI, Discovery MI-DR

## Abstract

**Background:**

This article compares the physical performance of the 4-ring digital Discovery MI (DMI) and PMT-based Discovery MI-DR (DMI-DR) PET/CT systems. Physical performance was assessed according to the NEMA NU 2-2012 standards. Performance measures included spatial resolution, image quality, scatter fraction and count rate performance, and sensitivity. Energy and timing resolutions were also measured. Published DMI and DMI-DR performance studies from other centers are reviewed and compared.

**Results:**

4-ring DMI spatial resolution at 1-cm radial offset in the radial, tangential and axial directions was 4.62, 4.18 and 4.57 mm, respectively, compared with the DMI-DR system values of 4.58, 4.52, and 5.31 mm. Measured sensitivity was 13.3 kcps/MBq at the center of the FOV and 13.4 kcps/MBq 10 cm off-center for the SiPM-based DMI system. DMI-DR system sensitivity was 6.3 kcps/MBq at the center of the FOV and 6.8 kcps/MBq at 10 cm off-center. DMI measured noise equivalent count rate peak was 175.6 kcps at 20.1 kBq/ml; DMI-DR was 146.7 kcps at 31.7 kBq/ml. Scatter fraction was 40.5% and 36.6%, respectively. DMI image contrast recovery (CR) values ranged from 73.2% (10 mm sphere) to 91.0% (37 mm sphere); DMI-DR, values ranged from 68.4% to 91.4%. DMI background variability (BV) was 1.8%–6.5%; DMI-DR was 2.3%–9.1%. The Q.Clear algorithm improved image quality, increasing CR and decreasing BV in both systems. The photopeak energy resolution was 9.63% and 12.19% for DMI and DMI-DR, respectively. The time-of-flight (TOF) resolution was 377.26 ps and 552.71 ps, respectively. Compared with measurements in other centers, results were similar and showed an absolute mean relative deviation of 6% for DMI and 7% for DMI-DR overall performance results.

**Conclusions:**

Performance measures were higher for the 4-ring DMI than the DMI-DR system. The biggest advantages of the 4-ring DMI vs DMI-DR are improved sensitivity and count rate performance. This should allow a better image signal-to-noise ratio (SNR) for the same acquisition times or, similar SNR with lower acquisition times or injected activity. In its 3-ring configuration, the DMI showed worse performance results than the PMT-based system in terms of count rate scatter fraction and image quality (for similar axial FOV).

## Background

Positron emission tomography/computed tomography (PET/CT) scanners have proven their role as multimodality medical imaging tools, and are now integrated into routine medical practice. General Electric (GE) Healthcare (Milwaukee WI, USA) recently launched the new digital Discovery MI (DMI) and PMT-based Discovery MI-Digital Ready (DMI-DR) whole body time-of-flight (TOF) PET systems. In an era when several different PET/CT scanners may be present in the same site or hospital, a thorough evaluation and comparison are important to optimize the use of all systems based on a deep knowledge of their similarities and differences.

National Electric Manufacturer’s Association/Association of Electrical Equipment and Medical Imaging Manufacturers (NEMA) performance measurements are rigorous tests performed to ensure that imaging systems are fully operational and behave according to specifications. Measurements are performed before system acceptance and serve as a reference for future tests to make sure that the PET performances has not degraded over time. The standard NEMA NU 2-2012 guidelines for PET [1] includes a series of tests for spatial resolution, image quality, scatter fraction, and count rate performance, accuracy of correction for count losses and random events, and sensitivity.

The purposes of this work were to evaluate the physical performances of the SiPM-based 4-ring DMI and PMT-based DMI-DR PET/CT systems according to these standards and to compare their performance using the NEMA measurements. In addition, a comparison of NEMA data at our site with publicly available data for the 4-ring DMI system at Stanford and Uppsala Universities [2] and Tokyo [3] will be provided. A comparison with the 3-ring DMI system installed at Brugge [4] will be also presented. For the DMI-DR system, previous work from Southampton-Poole-Plymouth [5] will be considered for comparison. Finally, a comparison with NEMA measurements performed for other commercially available PET/CT systems will be presented and discussed.

## Methods

### PET/CT systems

Discovery MI is the latest generation of PET/CT scanners commercialized by GE Healthcare and the first digital PET/CT system developed by the company. In early 2018, Hadassah-Hebrew University Medical Center received and installed the DMI PET/CT system. The GE DMI PET/CT system combines a 128-slice computed tomography (CT) system and a 4-ring PET system with LightBurst digital detectors providing a 20-cm axial field-of-view (AFOV) and a 70-cm transaxial field-of-view (TFOV). Each ring consists of 34 detectors, each of which contains four detector blocks, for a total of 544 detector blocks. Each detector block contains a 4 × 9 array of small lutetium-based scintillators (LBS), with crystals placed on three 3 × 2 arrays of silicon photomultiplier (SiPM) detectors, for a total of 19,584 crystals and 9792 SiPM channels. The SiPM signal readout electronics is implemented as an application-specific integrated circuit (ASIC). The output energy is digitized by an external analog-to-digital converter (ADC) and the timing signal by an external time-to-digital converter (TDC). Each crystal element is 3.95 mm × 5.3 mm × 25 mm, with several crystals connected to light guides that optimize light collection and improve sensitivity and resolution. The coincidence window is 4.9 ns, and the lower (LLD) and upper (ULD) level energy discriminators are 425 keV and 650 keV, respectively. These LBS crystals and SiPMs allow the DMI system to be TOF-capable with a timing resolution below 380 ps [2]. Moreover, integrated real-time digital temperature sensors, coupled with a closed liquid cooling system, maintain SiPM detector temperature at 18 °C to improve stability. Detector gain is adjusted in real-time as a function of temperature. The DMI system is also commercially available in a 3-ring configuration that provides a 15-cm AFOV.

A few months later, we also installed the GE DMI-DR system. The GE DMI-DR PET/CT system is a new PMT-based system with the capability to host the LightBurst Digital PET detectors combining the same 128-slice CT system. The DMI-DR AFOV and TFOV are equal to 15.6 cm and 70 cm, respectively. In this model, each ring consists of 24 detectors. There are a total number of 13,824 LBS crystals for the whole system, with a 4.2 mm × 6.3 mm × 25 mm dimension of each element. Here also, several crystals are connected to light guides. The total number of photomultiplier tubes is 1024. The coincidence window and the LLD and ULD are the same as for the DMI system. The timing resolution for the Discovery MI-DR is close to 550 ps [6]. Upgrade of DMI-DR to DMI involves replacement of the PET gantry (SiPMs instead of PMTs) but not of the CT gantry or stationary base. Additional requirements include adding non-gantry components such as a chiller, dehumidifier, power distribution unit, and a software update.

Both systems allow use of the same clinical reconstruction algorithms, namely VUE Point HD (VPHD), VUE Point FX (VPFX), and Q.Clear. VPHD is a 3D maximum likelihood ordered subset expectation maximization (3D OSEM) [7] image reconstruction algorithm that includes corrections for scatter, random events, dead time, and attenuation (from CT). VPFX is a VPHD algorithm that uses TOF information (3D OSEM + TOF) for better localization of the annihilation event, improving signal-to-noise ratio (SNR) in reconstructed images. VPHD and VPFX algorithms can also include point spread function (PSF) modeling to improve PET image contrast-to-noise ratio (CNR) [8], leading to their characterization as VPHD-S (3D OSEM + PSF) and VPFX-S (3D OSEM + TOF + PSF) reconstruction algorithms, respectively. The most recent Q.Clear algorithm is a Bayesian penalized-likelihood iterative image reconstruction algorithm [9, 10], where the sole user-input value, termed β, controls the relative strength of the noise regularizing term. A typical β value for ^18^F-FDG examinations is around 400 [10]. The advantage of such an algorithm, in contrast with OSEM, is that Q.Clear reaches full convergence [11] without the effects of excessive noise with increasing iterations, thereby maintaining good image quality.

### NEMA performance measurements

#### Spatial resolution

Spatial resolution measurements were performed according to the NEMA NU-2 2012 procedure [1] using a set of ^18^F point sources with a diameter of less than 1 mm radially and axially. The activity per source was about 1.5 MBq for the two systems. Point sources were inserted inside 75-mm-long glass capillary tubes (BRIS Micro-Hematocrit Capillary Tube, Thomas Scientific, Swedesboro, NJ, USA; soda-lime glass, 1.15 mm inner and 1.5 mm outer diameter, respectively). Measurements were performed with sources placed at 1, 10, and 20 cm radial offset vertically, and at axial positions z_0_ = 0 cm (CFOV) and z_1_ = 3/8 × AFOV offset from the center of the axial FOV (z_1_ = 7.5 cm for Discovery MI and z_1_ = 5.9 cm for Discovery MI-DR). Data were collected for 1 min at z_0_ and for 4 min at z_1_ to ensure similar counts in the two axial positions; at least 2 × 106 counts at each position. Acquired data were first reconstructed using the filter back projection (FBP) algorithm into a 384 × 384 × 71 matrix, without any filtering, as specified in the NEMA procedure [1]. Additional reconstructions were performed using the VPHD (3D OSEM) and VPHD-S (3D OSEM + PSF) algorithms in a 384 × 384 × 71 matrix with 4 iterations, 34 (DMI)/24 (DMI-DR) subsets, and a 2.0-mm Gaussian filter. Reconstruction parameters (matrix, number of iterations, subsets and filter value) were chosen according to “ideal” parameters specified in the “NEMA Test Procedures and Detector Performance test” brochures for DMI [12] and DMI-DR [13] provided by the vendor. Radial, tangential, and axial spatial resolutions at 1, 10, and 20 cm radial offset were averaged over the two axial positions and presented as the full width half maximum (FWHM) and the full width tenth maximum (FWTM) of the reconstructed PSF. Results were obtained using vendor-integrated software.

#### Image quality

The NEMA image quality test was established to provide an overall assessment of the imaging capabilities of the system under conditions resembling clinical scans. This test uses the NEMA IEC image quality body phantom (IQBP) (Model PET/IEC-BODY/P) [1], which contains spheres with an internal diameter of 10, 13, 17, 22, 28, and 37 mm and a 50-mm diameter cylindrical insert mounted in the center. The 10, 13, 17, and 22 mm spheres were filled with radioactive material (^18^F), while the two largest spheres held nonradioactive water. The lung insert provided with the phantom was filled with low-density material (polystyrene) and water. The 70-cm-long NEMA scatter phantom (Model PET/NEMA-SCT/P), used for count rate and scatter fraction performance measurements, was also used in this test. A line source inserted through the body of the scatter phantom was filled with a solution of radioactive ^18^F and water. The scatter phantom was placed adjacent to the IQBP and served as a source of out-of-field background activity in order to simulate clinical imaging conditions.

For the DMI system evaluation, the line source of the scatter phantom was filled with 99.2 MBq (2.7 mCi) of ^18^F, and the IQBP background region and spheres contained an ^18^F activity concentration of 6.37 kBq/mL (0.17 μCi/mL) and 25.48 kBq/mL (0.69 μCi/mL), respectively, at the time of the first acquisition.

For the DMI-DR evaluation, the phantom background region and spheres contained 5.6 kBq/mL (0.15 μCi/mL) and 22.4 kBq/mL (0.61 μCi/mL), respectively, at the time of the first acquisition. The line source contained 111.7 MBq (3.0 mCi) of ^18^F at that time.

In both cases, the IQBP was filled to reach a target-to-background ratio of 4:1. Three decay-adjusted acquisitions of 271, 279, and 282 s on DMI, and 212, 217, and 222 s on DMI-DR were performed, and data were corrected for random coincidences, normalization, dead-time losses, and scatter. A CT transmission scan (140 kV, auto mA) was acquired before the PET emission scans for attenuation correction. Data were reconstructed with the clinical VPHD (3D OSEM), VPHD-S (3D OSEM + PSF), VPFX (3D OSEM + TOF), and VPFX-S (3D OSEM + TOF + PSF) reconstruction algorithms in a 384 × 384 × 71 matrix, with 4 iterations, 34 (DMI)/24 (DMI-DR) subsets, and a 2-mm Gaussian filter. Here also, reconstruction parameters were chosen accordingly to [12, 13]. Q.Clear reconstructions with β = 50, 200, and 350 were also performed for comparison. A value of β = 350 corresponds to the value used in clinical studies, as recommended by the manufacturer.

Contrast recovery (CR) and background variability (BV) were quantified using the integrated vendor software. The average and standard deviation (SD) of CR and BV were calculated over the three acquisition scans.

#### Count rate performance and scatter fraction

To determine the count rate response of the detectors at different activity levels in the PET FOV and the number of scattered-to-total events, the cylindrical 70-cm-long scatter phantom was used according to NEMA procedure [1]. The phantom was positioned such that its center was aligned with the center of the FOV. Following vendor recommendations, a line source was filled with 819 MBq (22.1 mCi) of ^18^F for the DMI evaluation and with 1054 MBq (28.5 mCi) for the DMI-DR evaluation at the start of the acquisitions so that the peak count rate of the systems would be exceeded. The source was placed inside the phantom with a 4.5-cm vertical offset. Data were acquired for about 10 h and over a total of 24 frames (17 frames of 15 min followed by 7 frames of 25 min with a 25-min delay between consecutive frames). Prompts, noise equivalent count rate (NECR), trues, random and scatter events, and the scatter fraction were calculated for both PET/CT systems using the vendor-integrated software.

#### Accuracy of correction for count losses and random events

Data acquired for the count rate performance and scatter fraction tests reconstructed using the VPHD (3D OSEM) algorithm in a 128 × 128 matrix were used for this test. Count loss and random correction accuracy were calculated below the peak NEC by comparing the trues rates (i) calculated using count losses and random corrections, and (ii) the true rates extrapolated from measurements (least-square fit) with negligible count losses and random events. Mean, minimum, and maximum deviations were obtained using the vendor-integrated software and recorded for each system.

#### Sensitivity

The sensitivity test measures the coincidence detection rate of the system for a given amount of activity in the FOV. In accordance with NEMA specifications [1], sensitivity was measured using a 70-cm-long thin line filled with an amount of ^18^F activity low enough to minimize any dead time in the system: 2.51 MBq (68 μCi) for DMI and 2.80 MBq (76 μCi) for DMI-DR at the start of the acquisitions. In order to ensure that all positrons annihilate near the decay site, the line source was placed inside high-density material that stops all positrons. NEMA attenuation-free sensitivity in the air was obtained from successive 1 min measurements of the count rate using five aluminum sleeves of known thicknesses (phantom Model PET/NEMA-SEN/P). The data were exponentially extrapolated to zero sleeve thickness, as described first by Bailey et al. [14]. Measurements were performed at the center of the FOV and at a 10-cm horizontal offset. Data were analyzed using the vendor-integrated software after random subtraction to ensure true-only datasets. NEMA attenuation-free sensitivity in the air and the axial sensitivity profile at the center of the FOV and at a 10-cm radial offset were obtained with the vendor-integrated software.

All ^18^F activity measurements for phantom preparations were done using a Capintec CRC®-55tR dose calibrator, channel #472 (calibrated with respect to national standards).

### Energy and coincidence timing resolution

In addition, since the DMI and DMI-DR are TOF PET/CT systems, TOF performance was evaluated using another methodology. This last test also measured energy resolution for these two systems.

The timing resolution and energy resolution were evaluated using an experimental setup similar to the one in [15]. The 70-cm-long line source used during the sensitivity test was filled with 19.23 MBq (0.52 mCi) of ^18^F for DMI and 15.51 MBq (0.42 mCi) for DMI-DR. The line source was placed inside a single aluminum sleeve at the CFOV in the axial direction. A 30-min acquisition was performed, yielding a minimum of 300 million counts. Calculation of the timing resolution FWHM was based on the Coincidence Time Correction (CTC) procedure described in [16] by fitting the timing spectra peaks for each crystal pair (for each line of response). The energy resolution was computed for each crystal and at each block level for all the modules. The system energy/timing resolution were quantified as the average of FWHM energy/timing values for each block.

### Comparisons with other studies

The NEMA NU 2-2012 parameters measured for the 4-ring DMI system (AFOV = 20 cm) were compared with results obtained at Stanford and Uppsala Universities [2] and Tokyo [3] for the same system and with results from Brugge [4] obtained for the system in its 3-ring configuration (AFOV = 15 cm). For the DMI-DR system, results were compared with those obtained at Southampton-Poole-Plymouth [5].

In a second step, a comparison was made between results obtained in our study and those published for other commercially available systems from Siemens Healthcare, Erlangen, Germany (Biograph mCT Flow [17, 18], Biograph mMR PET/MR [18, 19] and digital Biograph Vision [20]), Philips Medical Systems, Eindhoven, The Netherlands (Ingenuity TF [21] and Vereos [22, 23]) and GE Healthcare, Milwaukee, WI, USA (Discovery IQ [24, 25] and SiPM-based Signa PET/MR [26]).

## Results

### System performance

#### Spatial resolution

The radial, tangential, and axial spatial resolution, averaged over the two axial positions *z*_0_ and *z*_1_, for a radial offset of 1, 10, and 20 cm of the point source in terms of FWHM and FWTM for DMI and DMI-DR are listed in Table 1. Data are presented for the FBP and the clinical 3D-OSEM reconstruction algorithms, with and without modeling of the PSF. It is noteworthy that the results for the two systems are similar with, for instance, a spatial resolution for DMI and DMI-DR of 4.62 vs 4.58 for FBP, 3.85 vs 4.13 for VPHD (3D OSEM) and 2.70 vs 2.84 for VPHD-S (3D OSEM + PSF) at 1-cm radial offset.

**Table 1:** Radial, tangential, and axial spatial resolutions in terms of full width half maximum (FWHM) and the full width tenth maximum (FWTM) in mm at 1, 10 and 20 cm radial offset for the Discovery MI and DMI-DR systems with different reconstruction algorithms (filter back projection (FBP), VPHD (3D OSEM), and VPHD-S (3D OSEM + PSF)

(mm)	FBP reconstruction algorithm				VPHD (3D OSEM) reconstruction algorithm				VPHD-S (3D OSEM + PSF) reconstruction algorithm			
	Discovery MI		Discovery MI-DR		Discovery MI		Discovery MI-DR		Discovery MI		Discovery MI-DR	
	FWHM	FWTM	FWHM	FWTM	FWHM	FWTM	FWHM	FWTM	FWHM	FWTM	FWHM	FWTM
1-cm radial offset												
Radial	4.62	8.95	4.58	8.31	3.85	7.63	4.13	8.00	2.70	5.08	2.84	5.15
Tangential	4.18	8.65	4.52	6.41	3.77	7.75	3.89	7.79	2.69	5.12	2.82	5.15
Axial	4.57	10.42	5.31	12.03	4.07	9.91	4.87	11.59	3.01	5.61	3.69	7.86
10-cm radial offset												
Radial	5.56	10.58	5.43	10.35	4.68	8.83	4.69	9.19	2.78	5.16	2.64	4.88
Tangential	4.69	9.32	4.90	9.47	3.91	7.87	3.98	8.04	2.77	5.15	2.72	5.05
Axial	6.05	11.70	5.58	12.04	4.92	9.12	4.37	10.30	4.15	7.51	3.50	6.84
20-cm radial offset												
Radial	7.39	14.23	7.16	13.04	7.26	12.78	6.83	12.22	3.14	5.79	3.00	5.60
Tangential	5.08	9.28	4.82	9.46	4.16	8.07	4.06	8.14	2.90	5.31	2.75	5.14
Axial	6.05	12.00	5.91	12.31	4.56	9.64	4.66	11.03	3.43	7.15	3.60	7.07

**Fig. 1:**
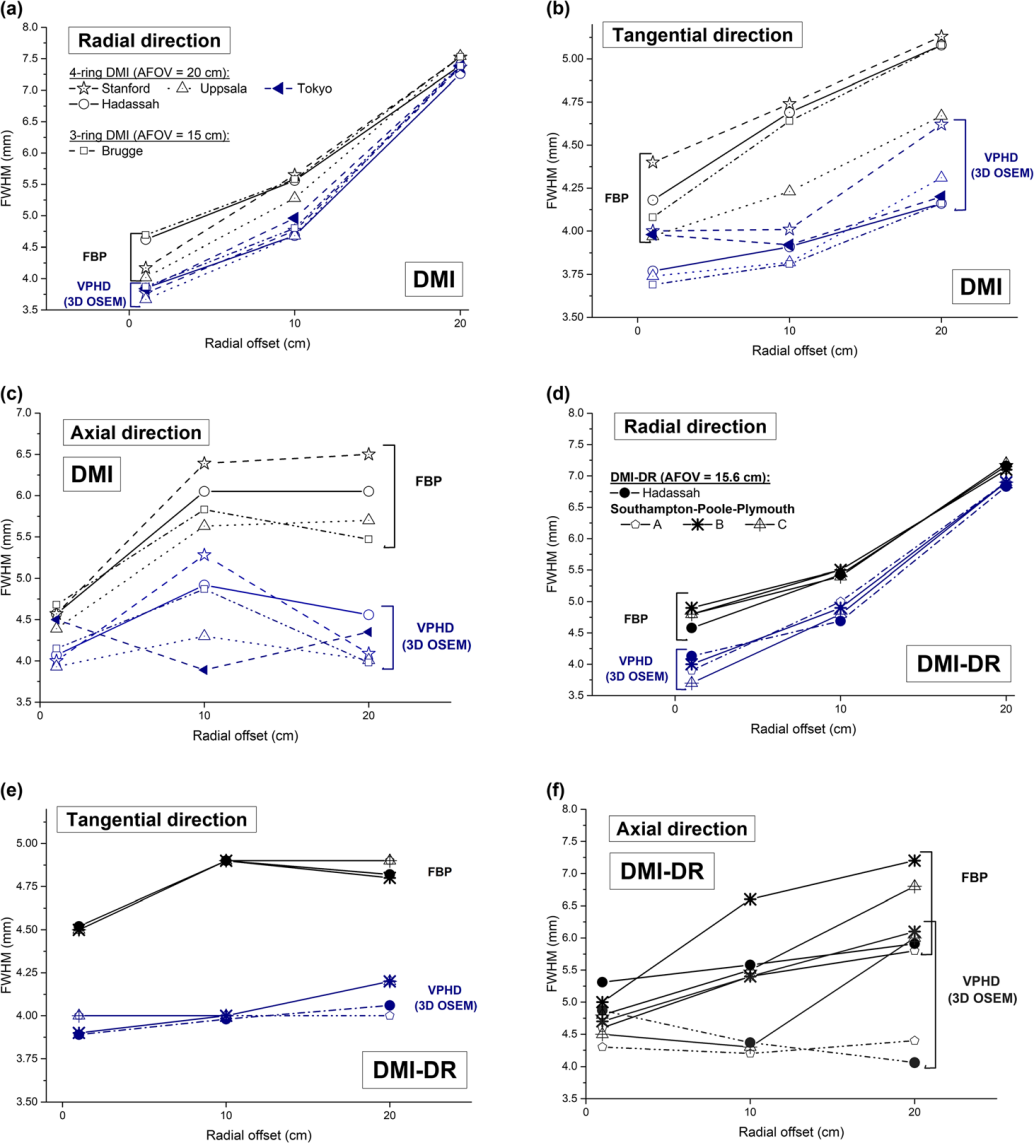
Radial, tangential, and axial spatial resolutions in mm (FWHM) at 1, 10, and 20 cm radial offset obtained a Hadassah for the **a**-**c** DMI and **d**-**f** DMI-DR systems using FBP and VPHD (3D OSEM) reconstruction algorithms and compared with results obtained in other centers [2–5]

For the DMI evaluation, the results obtained at our institution are compared with previous results from Stanford and Uppsala Universities [2], Tokyo [3] and Brugge [4] in Fig. 1a–c. The latter show that the spatial resolution from the DMI system across the five centers is very similar, regardless of the 3-ring or 4-ring configuration.

For DMI-DR, Fig. 1d–f also shows comparable results across the different centers.

#### Image quality

**Fig. 2:**
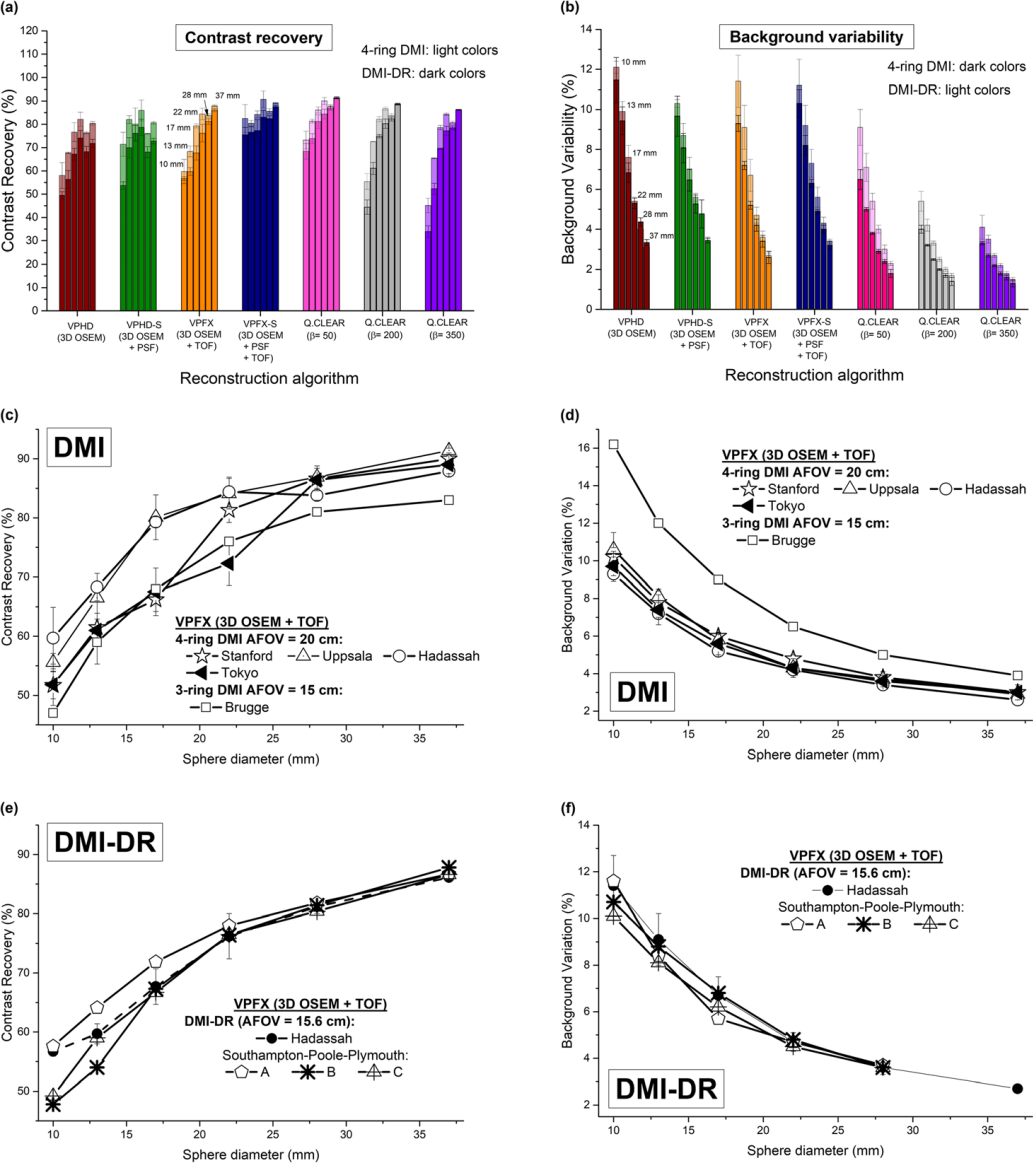
**a** Contrast recovery (%) and **b** background variability (%) for Discovery MI, and Discovery MI-DR PET/CT systems using VPHD (3D OSEM), VPHD-S (3D OSEM + PSF), VPFX (3D OSEM + TOF), VPFX-S (3D OSEM + TOF + PSF) reconstruction algorithms and Q.Clear with β = 50, 200, and 350. Results obtained at Hadassah for VPFX reconstruction algorithms are compared to Stanford and Uppsala [2], Tokyo [3] and Brugge [4] measurements for DMI (**c**-**d**) and to Southampton-Poole-Plymouth [5] for DMI-DR (**e**-**f**)

CR and BV obtained from images acquired using the NEMA NU 2-2012 IQBP phantom and reconstructed using the VPHD (3D OSEM), VPHD-S (3D OSEM + PSF), VPFX (3D OSEM + TOF), VPFX-S (3D OSEM + TOF + PSF) and Q.Clear (β = 50, 200, 350) algorithms are shown in Fig. 2a and b for DMI and DMI-DR systems. For all reconstruction algorithms, better CR and BV results were obtained for DMI compared with DMI-DR, with a mean minimum/maximum relative difference of 4.6% (QC β = 50)/12.5% (VPHD) for CR, and 1.5% (VPHD)/35.8% (QC β = 50) for BV.

For both systems, the use of PSF increased hot sphere, but not cold sphere contrast. The addition of the TOF information to PSF resulted in an overall improvement in CR and BV. The Q.Clear algorithm improved the image quality by further decreasing the BV. Indeed, the VPFX-S and QC (β = 50) reconstruction algorithms presented a similar CR (slightly higher for QC), but a lower BV for QC. It is noteworthy that CR and BV decreased with increasing values of the β parameter although all QC results remained similar.

Figure 2c, d presents the results obtained for the DMI system at Stanford and Uppsala Universities [2], Tokyo [3] and Brugge [4] in comparison with the results obtained at our institution using the VPFX algorithm. It is noteworthy that image quality results are very similar across centers, apart from the BV obtained at Brugge for the 3-ring system, with a minimum/maximum relative deviation of 74%/50% from our results is obtained.

Figure 2e, f shows similar image quality results obtained at Southampton-Poole-Plymouth [5] and our center for the DMI-DR system.

**Fig. 3:**
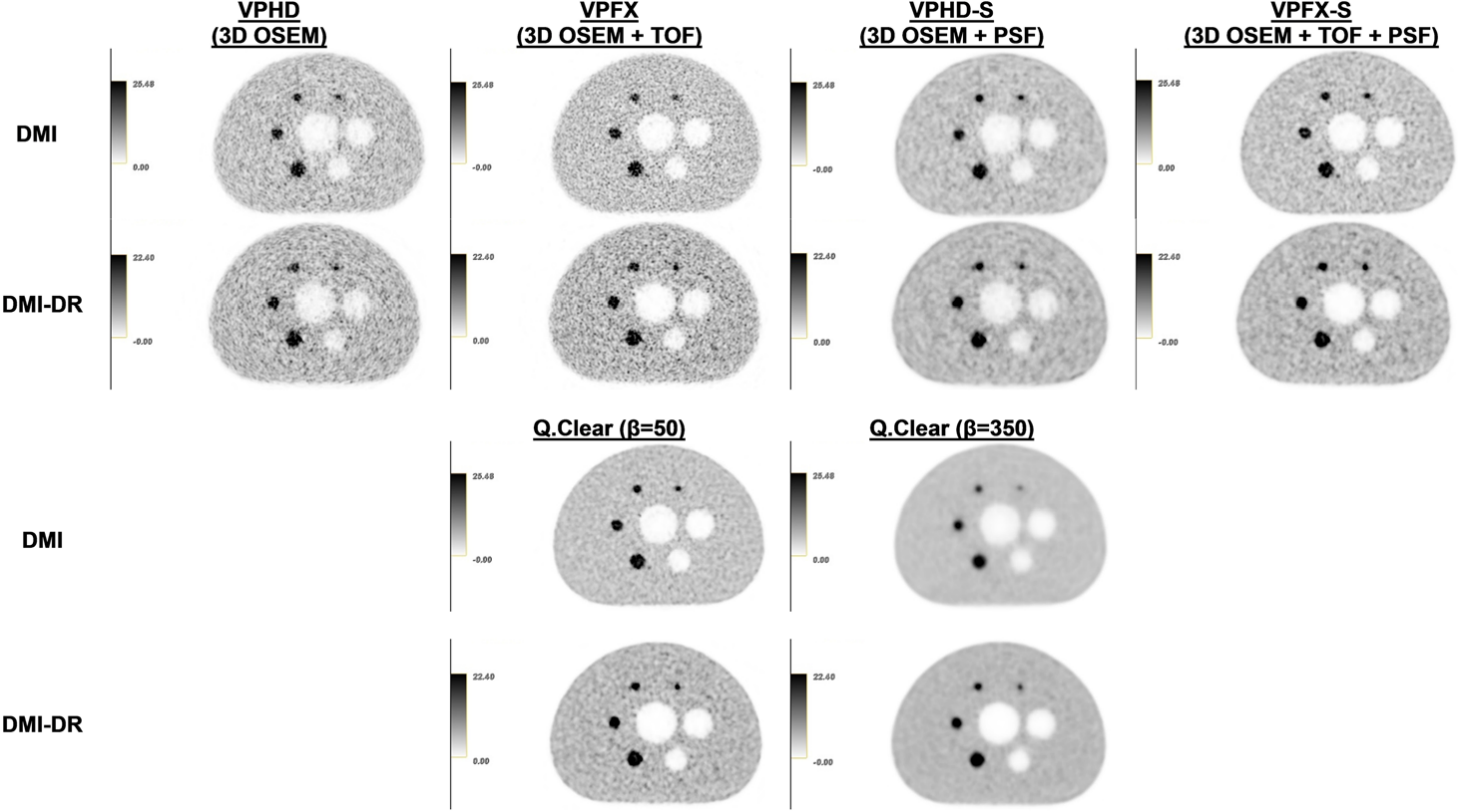
Central slices of the NEMA IEC image quality body phantom acquired during the image quality tests for the Discovery MI and Discovery MI-DR PET/CT systems. Images were reconstructed with the VPHD (3D OSEM), VPFX (3D OSEM + TOF), VPHD-S (3D OSEM + PSF), VPFX-S (3D OSEM + TOF + PSF) reconstruction algorithms and with Q.Clear, with β = 50 and β = 350. The gray-scale next to each phantom image represents the activity concentration in kBq/mL

Figure 3 presents the transverse views of the phantom acquisitions for the VPDH, VPFX, VPHD-S, VPFX-S, and Q.Clear (β = 50, 350) algorithms. A visual appreciation of the phantom acquisitions confirms the previous observations. The images illustrate better quality when using TOF information for the two PET/CT systems.

#### Count rate performance and the scatter fraction

**Fig. 4:**
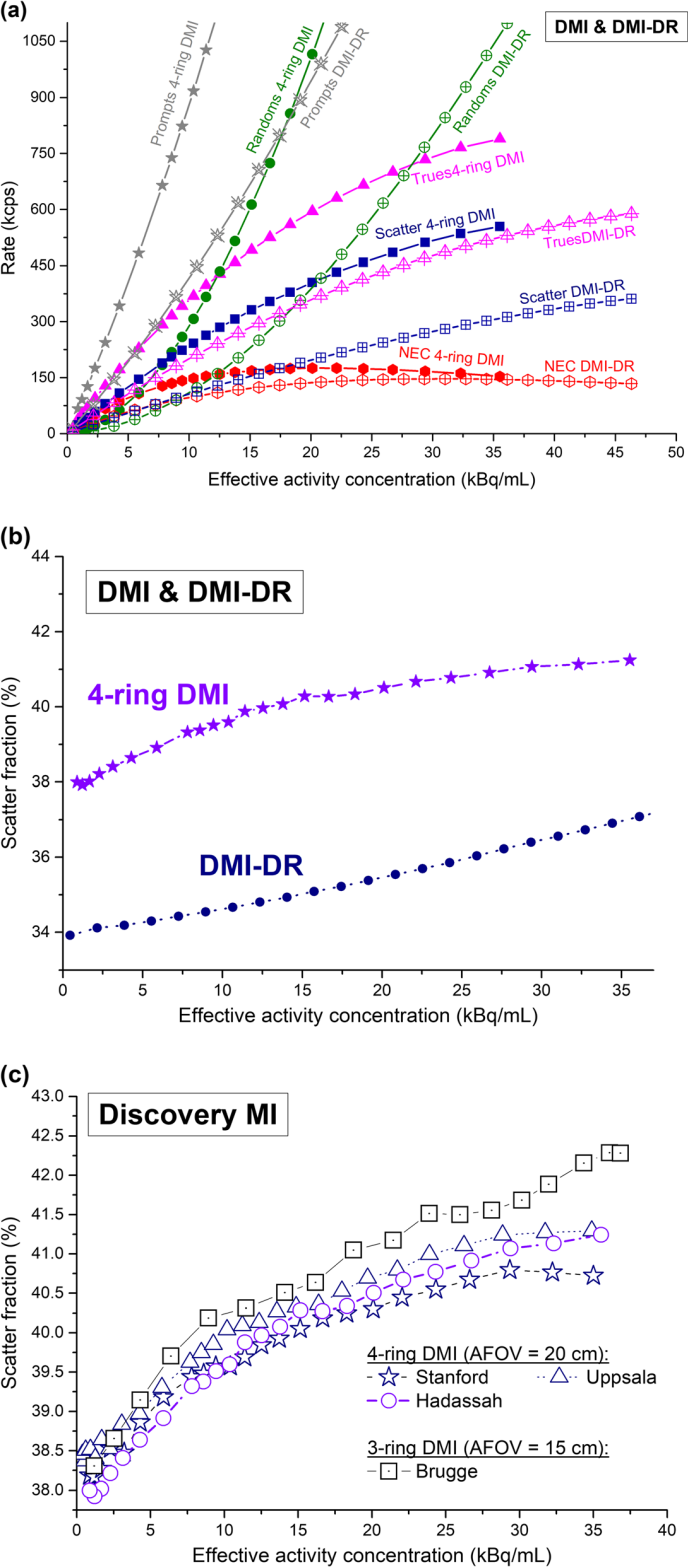
Count rate performance (prompts, NECR, true, random and scatter values) in kcps (**a**) and scatter fraction (**b**), versus the effective activity concentration in kBq/mL for the Discovery MI and Discovery MI-DR PET/CT systems at Hadassah. Comparison of the scatter fraction obtained at Hadassah, Stanford, and Uppsala [2] and Brugge [4] for the DMI system is presented in (**c**)

Figure 4a and b shows, respectively, the count rate performance and the scatter fraction of the DMI and DMI-DR systems. The peak NECR is 175.6 kcps at 20.1 kBq for DMI while, for DMI-DR, its value reaches 146.7 kcps at 31.7 kBq. At clinical activity concentrations, i.e., at about 5 kBq/mL, the NECR is 95.3 kcps for DMI and 58.5 kcps for DMI-DR. The scatter fraction of the two systems was slightly different over the range of activity concentrations, with values of 40.5% vs 36.5% at peak NECR, and 38.7% vs 34.3 % at 5 kBq/mL, for the DMI and DMI-DR systems, respectively.

Figure 4c compares the DMI scatter fraction to results from Stanford and Uppsala Universities [2], Tokyo [3] and Brugge [4]. Table 2 summarizes the count rate and scatter fraction results obtained for the two systems at different sites.

**Table 2:** Peak noise equivalent count rate (NECR), scatter fraction, peak true count rate and their corresponding activity concentration for the DMI-DR and DMI systems. Results obtained for DMI-DR at Southampton, Poole, and Plymouth (scanners A, B, C) [5] and for DMI at Stanford and Uppsala Universities [2], Tokyo [3] and Brugge [4] are listed for comparison

Measurement	MI-DR				MI				
	AFOV = 15.6 cm				AFOV = 20 cm				AFOV = 15 cm
	Present work	A	B	C	Present work	Stanford	Uppsala	Tokyo	Brugge
Peak NECR (kcps)	146.7	144	139	142	175.6	201.1	185.7	185.6	102.3
Activity at peak NECR (kBq/mL)	31.7	27.3	30.7	27.7	20.1	22.1	21.7	22.5	23.0
Scatter fraction at peak NECR (%)	36.6	36.8	37.5	36.9	40.5	40.4	40.8	42.1	41.2
Peak true count rate (kcps)	589.1				789.2	875.9	827.0		463.1
Activity at peak true count rate (kBq/mL)	46.3				35.5	35.4	34.8		36.9

#### Accuracy of the corrections for count losses and random events

The mean error for count rate accuracy was similar for DMI (3.11%) and DMI-DR (3.23%) at peak NECRs. For activity concentration in the clinical range, the mean error was 0.6% for DMI and 1.0% for DMI-DR.

#### Sensitivity

**Fig. 5:**
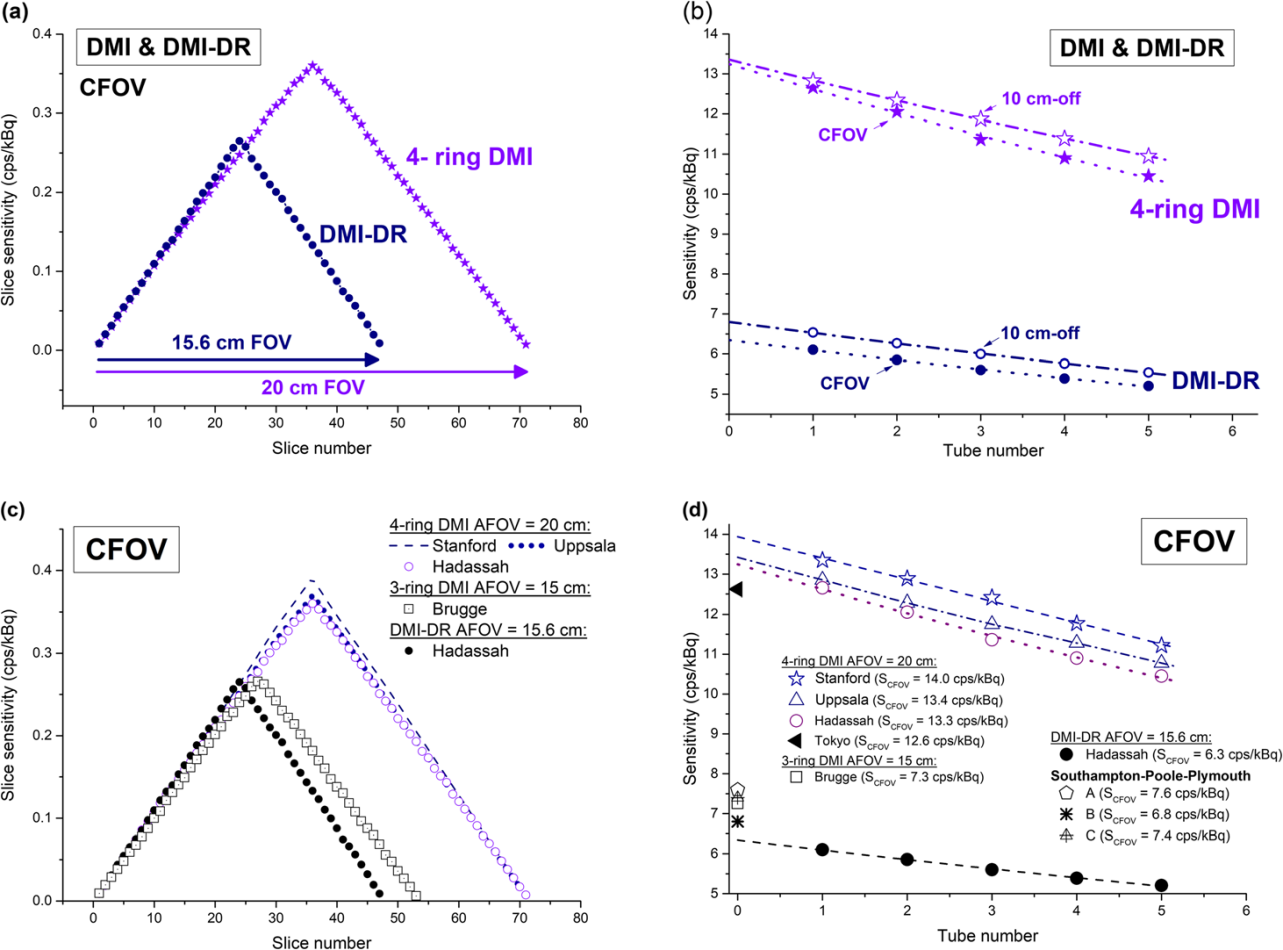
**a** Slice sensitivity profile at CFOV for DMI and DMI-DR. **b** Successive measurements of the count rate at CFOV and 10-cm radial offset using five aluminum sleeves and an extrapolation process to get the NEMA attenuation-free sensitivity in air. Comparison of the sensitivity results for the DMI and DMI-DR systems with results obtained in other centers [2–5] is presented in figures (**c**) and (**d**)

The sensitivity at CFOV for the digital DMI system was 13.3 cps/kBq and 13.4 cps/kBq at 10-cm radial offset. For the DMI-DR system, the sensitivity was 6.3 cps/kBq and 6.8 cps/kBq, respectively. Figure 5 shows (a) the slice sensitivity profile at CFOV and (b) the sensitivity at CFOV and at 10-cm radial offset for five aluminum sleeve thickness with the extrapolation of the NEMA attenuation-free sensitivity in the air (without attenuation material) for the 4-ring DMI and DMI-DR systems.

The results obtained at Uppsala and Stanford Universities [2] and Tokyo [3] for the 4-ring DMI system were 13.4 cps/kBq, 14.0 cps/kBq and 12.6 cps/kBq at CFOV, respectively. For the 3-ring DMI system, a sensitivity at CFOV of 7.3 cps/kBq was obtained at Brugge [4].

For the DMI-DR system, sensitivities at CFOV of 7.6 cps/kBq, 6.8 cps/kBq and 7.4 cps/kBq were obtained at Southampton-Poole-Plymouth [5].

Figure 5c and d shows the NEMA sensitivity results obtained in the different centers for DMI and DMI-DR.

### Energy and coincidence timing resolution

The system average energy resolution was 9.63% ± 0.08% for DMI and 12.19% ± 0.09% for DMI-DR in terms of FWHM. The system average timing resolution was 377.26 ± 2.62 ps for DMI and 552.71 ± 6.67 ps for DMI-DR.

At Stanford and Uppsala [2], photopeak energy resolution was 9.44 ± 0.07% and 9.35% ± 0.05% and the timing resolution was 374.16 ± 2.60 ps and 376.76 ± 2.70 ps, respectively. At Brugge [4] energy resolution was 9.30 ± 0.06% and the timing resolution was 375.60 ± 2.70 ps.

No energy or timing resolution data was available for the DMI system at Tokyo [3] and for the DMI-DR system at Southampton-Poole-Plymouth [5].

### Performance of other PET/CT systems

**Fig. 6:**
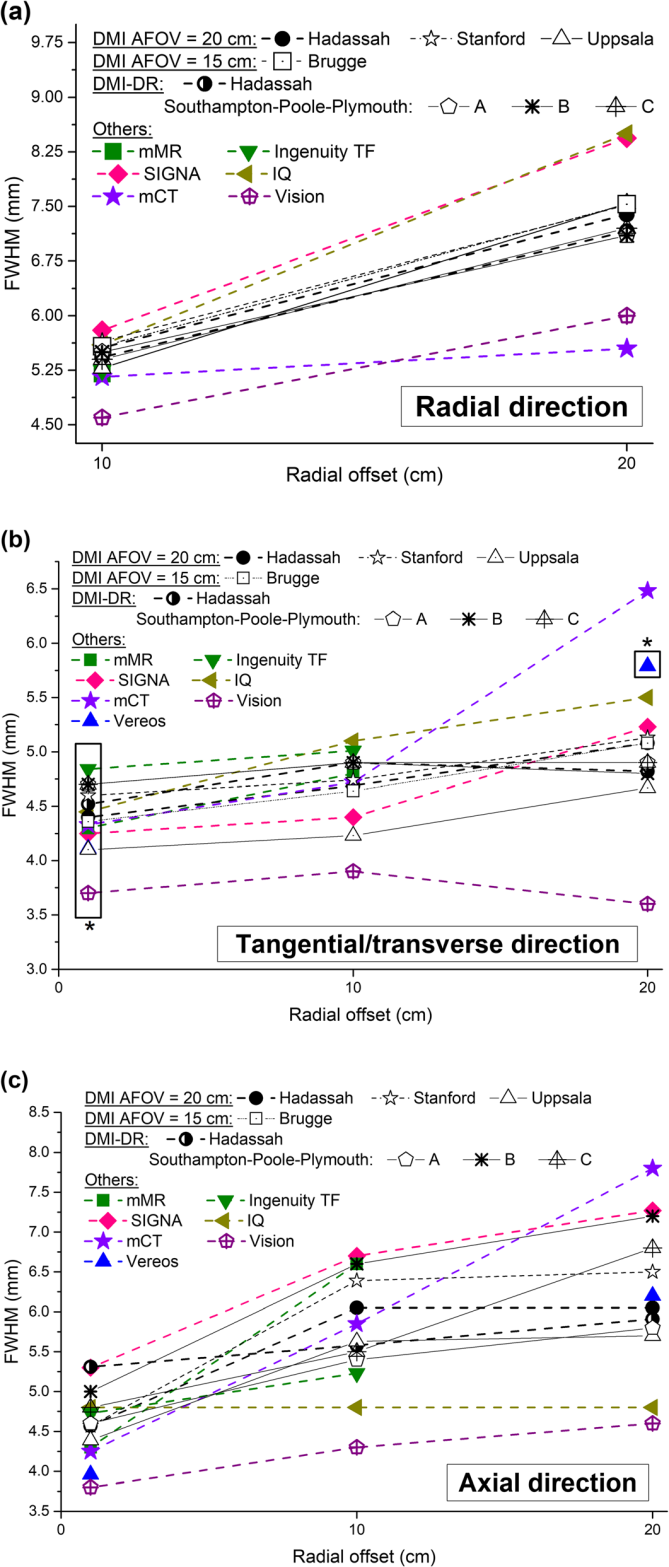
Resolution in mm (FWHM) for DMI, DMI-DR, Siemens Biograph mCT Flow [17, 18], Philips Ingenuity TF [21], Philips Vereos [22, 23], GE Discovery IQ [24, 25], GE SIGNA [26], Siemens Biograph mMR [18, 19] and digital Biograph Vision [20] systems in the **a** radial, **b** tangential or transverse (box with star) and **c** axial directions

**Fig. 7:**
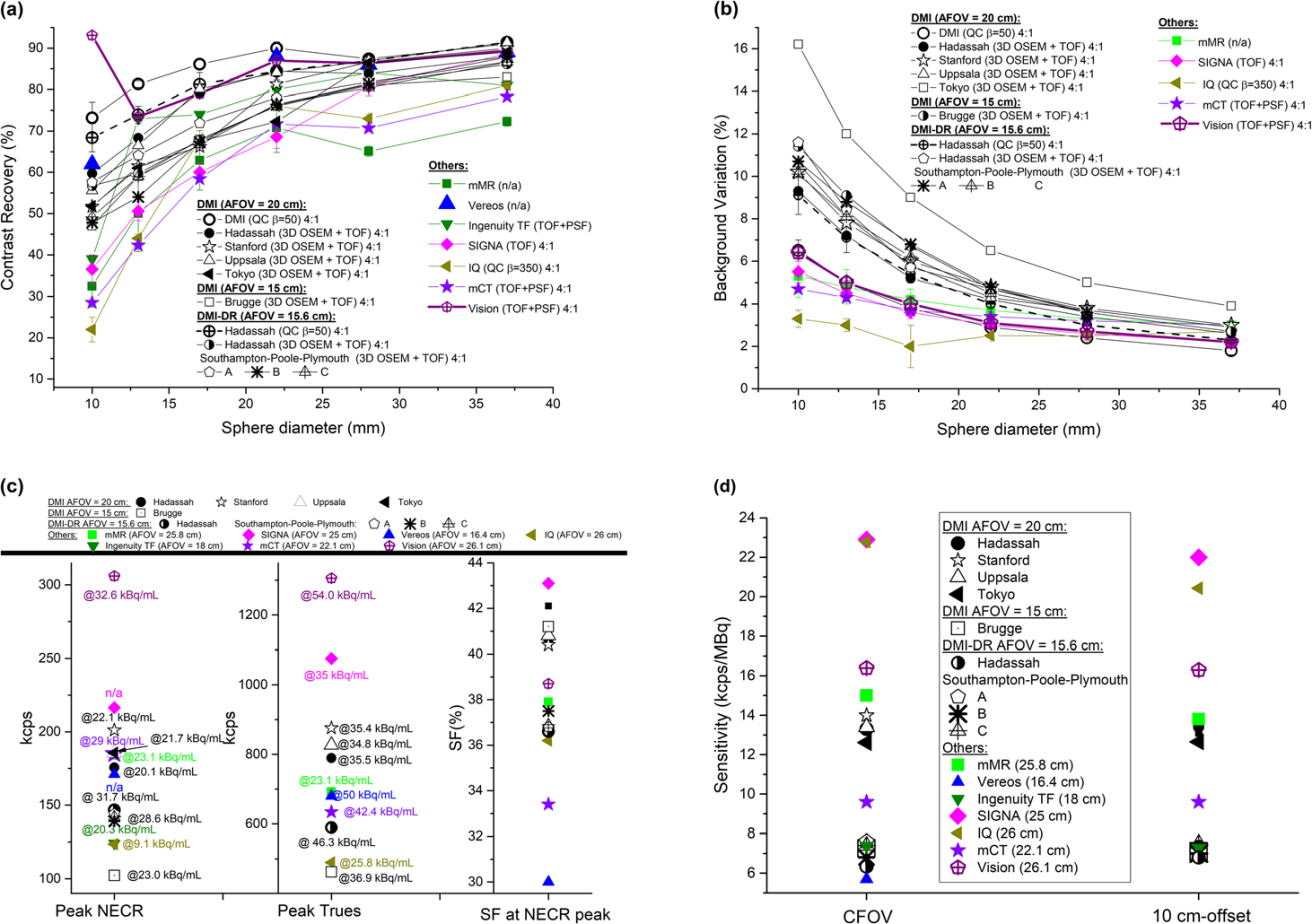
**a** Contrast recovery (%), **b** background variability (%), **c** peak NECR, peak trues, scatter fraction at NECR peak, and corresponding activity concentrations and **d** sensitivity at CFOV and 10-cm radial offset for DMI, DMI-DR, Siemens Biograph mCT Flow [17, 18], Philips Ingenuity TF [21], Philips Vereos [22, 23], GE Discovery IQ [24, 25], GE SIGNA [26], Siemens Biograph mMR [18, 19] and digital Biograph Vision [20]

Figures 6 and 7 present a comparison of previously published performance data for PET systems commercialized by Siemens Healthcare, Philips Medical Systems and GE Healthcare, with our findings.

Figure 6 shows the spatial resolutions at 1 cm [2, 17, 19–21, 23, 24, 26], at 10 cm [2, 17, 19–21, 24, 26], and 20 cm [2, 17, 20, 24, 26] radial offset in the (a) radial, (b) tangential/transverse, and (c) axial directions for the different systems. Figure 7a and b presents an appreciable comparison of the CR and BV results for the different systems. Count rate performances and scatter fraction for the different systems are presented in Fig. 7c. Finally, sensitivities at CFOV and at 10-cm-offset are presented in Fig. 7d.

## Discussion

In this work, the physical performance of the GE Healthcare 4-ring DMI and DMI-DR systems at our Medical Center were measured using the NEMA guidelines for system evaluation. It is noteworthy that, except for their similar spatial resolution (mean relative deviation of 0.9%, 1.6%, and 0.6% using FBP, VPHD (3D OSEM), VPHD-S (3D OSEM + PSF), respectively), performance of the 4-ring DMI system surpassed that of DMI-DR system. A similar spatial resolution between the two systems is expected due to very similar LBS crystal dimensions and to the fact that crystal thickness is one of the main contributors to the intrinsic response function of the system.

TOF performance was better for the digital 4-ring DMI system, which showed a timing coincidence resolution of 377.3 ps compared with 552.7 ps for the DMI-DR system. This result reflects the better timing performance of SiPM photodetectors compared with PMTs.

Sensitivity of the digital system in its 20 cm AFOV configuration (13.25 cps/kBq) was about twice that of the PMT-based PET/CT (6.34 cps/kBq), although a major contribution to the superior sensitivity of the DMI system is due to the difference in its extended AFOV (DMI-DR AFOV: 15.6 cm). In principle, the doubled sensitivity of the DMI system should allow for the maintenance of SNR with reduced acquisition times or injected activity of the radiopharmaceutical (by a factor of two). Alternatively, the image SNR should be enhanced using the same acquisition time. True values and NECR peaks are 25% and 16% higher for the 4-ring DMI system compared with the DMI-DR. At clinical concentrations, the differences in peak true values and NECR reach 46% and 37%, respectively, in favor of the 4-ring DMI system. Figure 4b shows that scatter fraction tends to increase with activity concentration, even though the attenuation/scatter distribution does not change, likely because of the pulse pile-up effect in the detectors. Indeed, if the detection of a 511 keV photon is confounded with a scattered photon of energy less than the lower energy threshold, pile-up may lead to an energy signal that lies within the energy window. Therefore, the detection probability for this scattered photon and the measured scatter fraction increases as activity increases [27, 28].

Image quality was also better for 4-ring DMI for all reconstruction algorithms. The biggest difference, in contrast, was found when reconstructing images with the VPHD (3D OSEM) algorithm (12.5%), while the difference decreased to its minimum (4.6%) when using the QC algorithm with β = 50. For background variability, the maximum difference is found for QC with β = 50 (35.8%) and the minimum for VPHD (1.5%). Comparing the conventional 3D-OSEM (PSF+TOF) and Q.Clear algorithms revealed an improvement in PET image quality, with a higher coefficient of recovery and lower background variability using Q.Clear. Indeed, it has been previously shown that most Q.Clear reconstructions improve CR and BV compared with OSEM depending on the β-factor [7, 26]. The increased CR with the Q.Clear reconstruction algorithm can be partly accounted for by the improved effective convergence of this algorithm. Also, the overall image quality using Q.Clear has been investigated and found to be the best reconstruction in most cases [26, 27], leading to a smooth and homogeneous appearance of background structures. Therefore, the use of Q.Clear can have implications for the assessment and quantification of treatment, especially in the detection of small foci. However, the optimal choice of β in Q.Clear has been shown to be challenging [26]. Indeed, the β-value depends on many factors such as the isotope used, the injected dose, the acquisition time per bed position, etc. All these factors need to be considered and harmonized for an optimal integration of Q.Clear in clinical PET/CT imaging.

Figure 3 presents transverse views of the phantom and highlights Gibbs ringing artifacts for the different reconstructions. An overshoot of this artifact mainly influences the CR, with higher CRs for the 22 mm sphere than for the 28 mm sphere as observed in Fig. 2a for the different reconstruction algorithms and for the two systems. It is noteworthy that the influence of the Gibbs artifact seems to be more significant for the DMI system than for DMI-DR.

This study included a comparison between the 4-ring DMI performance results obtained in our institution with those from Stanford and Uppsala Universities [2] and Tokyo [3] for the same system and with Brugge [5] for the 3-ring DMI system. The results obtained in the present paper are in close agreement with these previous results, which is reassuring. For spatial resolution (Fig. 1), the present measurements show a similar behavior with a minimum mean relative deviation of −1.17% for FBP from Brugge results and of 0.09% for VPHD (3D OSEM) from Tokyo. A maximum mean relative deviations of −6.17%/−3.31% for FBP/VPHD were obtained compared with Uppsala results. The mean deviation ± SD over all the measurements is −1.24 ± 5.98%. Image quality results for the 4-ring DMI system were also similar across all four centers (Hadassah, Uppsala, Stanford, Tokyo). Our findings show a minimum mean relative deviation of −0.2% in CR from Uppsala and 6.4% in BV from Tokyo. Maximum mean relative deviations in CR/BV of −8.1%/12.5% were found compared with Tokyo/Stanford. The 3-ring DMI system presented mean relative deviations of −11.3%/61% in CR/BV from our results. Compared with the DMI-DR results, the 3-ring DMI showed, on average, lower CR by 3.6% and higher BV by 38.3%. The peak NECR obtained in this work differed by only 5% from Uppsala and Tokyo and by 13% from Stanford. Compared with the 3-ring system, a much higher relative deviation of 42% is obtained. Compared with the DMI-DR system with a similar AFOV, the 3-ring DMI peak NECR is lower by 43%. Around clinical activity concentrations, i.e., 5 kBq/mL, NECR results obtained at Uppsala presented a relative deviation below 1%. Scatter fraction results were also very similar for all activity concentrations across the Hadassah, Uppsala, and Stanford centers with less than 0.6% maximum deviation. From Brugge results, it is noteworthy that the scatter fraction for the DMI system in its shorter 15 cm AFOV configuration is similar or even higher to that of the 4-ring system. One might think that the scatter fraction observed at NECR peak for the 4-ring DMI was higher than DMI-DR (Fig. 4b) mainly due to its larger AFOV, keeping in mind that the energy acceptance windows are the same and energy resolutions are similar for the two systems. However, it is notable that for similar AFOV, the scatter fraction of the 3-ring DMI system is higher than the DMI-DR by about 5% in average. Sensitivity for our system at CFOV differed by 0.7% from Uppsala and by 5% from Stanford and Tokyo results. The CFOV sensitivity for the 3-ring DMI system obtained at Brugge was 45% lower than the 4-ring system and similar to that of the PMT-based DMI-DR system.

In view of the NEMA data of the 3-ring DMI (15 cm AFOV) and of the DMI-DR (15.6 cm AFOV) systems, it seems that the SiPMs LightBurst Digital Detectors do not present a real advantage compared with standard PMTs.

This work included also a comparison between four DMI-DR systems. Performance results obtained in the various sites differed from our findings by 2.8 ± 11.4% in spatial resolution, −2.4 ± 5.8% in image quality, −3.4 ± 1.4% in peak NECR, and 15.3 ± 5.4% in CFOV sensitivity. As specified by Tsu et al. [2], variations in the system manufacturing process may explain general trends in the variation between systems. Moreover, variations in activity calibrations used in the dose calibrator may also cause differences in performance results between the different systems.

Finally, the study presented a comparison of the DMI and DMI-DR system performance with that of other commercially available systems. We can note from Fig. 6 that spatial resolution results of the different PET systems commercialized by GE healthcare are very similar. However, the Siemens Biograph Vision seems to outperform all its competitors. The PET scanner with the narrowest crystal dimensions is the Vision system. Decreasing the crystal dimensions tends to improve the spatial resolution due to less light spread in the crystal. On the other hand, increasing the crystal thickness improves the sensitivity of the system. Indeed, for similar AFOV, the GE systems (DIQ and SIGNA) present thicker crystals and better sensitivity.

As specified for the DMI and DMI-DR systems, the TOF performance of the SiPM-based systems (Vereos, SIGNA, and Vision) is better than for the PMT-based PET/CT systems (Ingenuity TF, mCT). The Siemens mMR system lacks TOF capability due to the relatively low timing resolution of avalanche photodiodes (APDs). The GE DIQ uses BGO as the scintillating crystal and also lacks TOF capability.

Image quality results cannot be compared easily. Indeed, NEMA NU-2 2007 standards included longer acquisitions than the acquisition times specified in the NEMA NU-2 2012 standards, leading to favorable CR and BV in measurements using the earlier standards. Tests of the Biograph mMR PET/MR (Siemens Healthcare) [19] and the Ingenuity TF (Philips Medical Systems) [21] were performed according to the NEMA NU-2 2007 standards. Moreover, reconstruction algorithms are not the same for all the systems. From Fig. 7a and b, it is noteworthy that using the Q.Clear algorithm with β = 50 gives the best results for the DMI and DMI-DR systems with with CR coefficients remaining at a constant high level (>70%) across all sphere diameters. Results of the Siemens Biograph Vision are very close to those of DMI and DMI-DR (Q.Clear). The very high contrast recovery obtained in [20] for the smallest sphere is due to the Gibbs artifact when using PSF, as specified there. The influence of this artifact can also be observed for the 22 mm sphere in the Vision, DIQ, mCT and mMR systems on Fig. 7a. Moreover, it should be pointed out that although the 3-ring DMI gives intermediate CR values, the BV values are higher than its competitors.

The count rate results for the Biograph Vision system (AFOV = 26.1 cm) were the best of all PET/CT systems compared in this study (Fig. 7c), with NECR higher by 52% compared with the DMI (GE Healthcare) system (AFOV = 20 cm) and by 145% compared with DIQ (GE Healthcare) system (AFOV = 26 cm) or the Ingenuity TF (AFOV = 18 cm). The DMI NECR results were similar compared with the mCT (AFOV = 22.1 cm) and mMR (AFOV = 25.8 cm) systems with a larger AFOV. For the DMI-DR system, (AFOV = 15.6 cm), peak NECR was higher by 15% compared with the DIQ or Ingenuity TF systems, but below other systems. The worse count rate results were obtained for the 3-ring DMI system, with NECR lower by about 200% compared with Biograph Vision and by 67% compared with Vereos (with a similar AFOV). We should note that the higher photon light output and faster decay time of LSO/LYSO crystals allow for higher count rates and result in a higher NECR value compared with BGO. However, the BGO detector does have better sensitivity than LYSO due to its better stopping power.

As shown in Fig. 7d, with the exception of the DIQ and Biograph Vision with their larger AFOV, the DMI system has the highest sensitivity compared with other PET/CT systems. The AFOV of the Biograph Vision (26.1 cm) is larger by about 30% compared with the DMI, leading to a similar level of sensitivity improvement. However, the DIQ, with a similar AFOV (26 cm), shows a sensitivity 74% higher than the DMI.

Sensitivity of the DMI-DR system is similar to that of the digital Philips Vereos PET/CT system, which has a similar AFOV (DMI-DR: 15.6 cm; Vereos: 16.4 cm).

There are several limitations to our study. First, the NEMA NU 2-2012 analysis described in the present paper used the vendor-supplied software. As such, the work is not a completely independent assessment of performance. The development of homemade software for analysis would have added greater value to this paper. Also, the TOF performance of the scanners was not evaluated using the NEMA NU-2 2018 [29, 30] standards, where timing resolution has to be described as a function of the effective radioactivity concentration. Timing resolution is mainly degraded by pile-up, which varies with activity concentration.

## Conclusion

According to measurements based on NEMA NU-2 2012 standards, the 4-ring digital DMI (AFOV = 20 cm) presents overall better performance compared with the PMT-based DMI-DR (AFOV = 15.6 cm) PET/CT scanner, most notably with sensitivity approximately doubled. However, the Discovery MI system, in its 15 cm AFOV configuration, presents worse performance than the PMT-based system in terms of count rate, scatter fraction, and image quality. Improved image quality is obtained with the Q.Clear reconstruction algorithm, with increased CR and decreased BV. Finally, compared with other PMT-based PET/CT systems with similar or higher AFOV, the 4-ring DMI system with its LightBurst Digital Detectors offers better overall performance characteristics.

## Data Availability

NEMA raw data images are stored and archived on a CD at the Hadassah-Hebrew University Medical Center, Jerusalem, Israel.

## Abbreviations

ADC: Analog-to-digital converter
AFOV: Axial field-of-view
APDs: Avalanche photodiodes
ASIC: Application-specific integrated circuit
BV: Background variability
CFOV: Center of the field-of-view
CNR: Contrast-to-noise ratio
CR: Contrast recovery
DMI: Discovery MI
DMI-DR: Discovery MI-Digital Ready
FBP: Filtered back projection
FWHM: Full width half maximum
FWTM: Full width tenth maximum
GE: General Electric
IQBP: Image quality body phantom
LBS: Lutetium-based scintillators
LLD: Lower-level energy discriminators
NECR: Noise equivalent count rate
NEMA: National Electric Manufacturer’s Association
OSEM: Ordered subsets expectation maximisation
PET/CT: Positron emission tomography/computed tomography
PMT: Photomultiplier tube
PSF: Point spread function
QC: Q.Clear
SD: Standard deviation
SiPM: Silicon photomultiplier
SNR: Signal-to-noise ratio
TDC: Time-to-digital converter
TFOV: Transaxial field-of-view
TOF: Time-of-flight
ULD: Upper-level energy discriminators
VPFX: VUE Point FX
VPHD: VUE Point HD
